# IFITM1, CD10, SMA, and h-caldesmon as a helpful combination in differential diagnosis between endometrial stromal tumor and cellular leiomyoma

**DOI:** 10.1186/s12885-021-08781-w

**Published:** 2021-09-23

**Authors:** Weilin Zhao, Mei Cui, Ruiqi Zhang, Xihua Shen, Xin Xiong, Xinhua Ji, Lin Tao, Wei Jia, Lijuan Pang, Zhenzhu Sun, Chun Wang, Hong Zou

**Affiliations:** 1 Department of Pathology, The First Affiliated Hospital, Shihezi University School of Medicine, Key Laboratory of Xinjiang Endemic and Ethnic Diseases of the Ministry of Education of China, NHC Key Laboratory of Prevention and Treatment of Central Asia High Incidence Diseases, Xinjiang, 832002 China; 2 Department of Pathology, Taihe Hospital, Hubei University of Medicine, Shiyan, 442000 Hubei China; 3 Department of Pathology, Xinjiang Uygur Autonomous Region People’s Hospital, Xinjiang, 830001 China; 4 Department of Pathology, The Second Affiliated Hospital of Zhejiang University School of Medicine, Zhejiang, 310009 China

**Keywords:** IFITM1, CD10, SMA, H-caldesmon, Endometrial stromal tumor, Cellular leiomyoma

## Abstract

**Background:**

The differential diagnosis of endometrial stromal tumor (EST) and uterine cellular leiomyoma (CL) remains a challenge in clinical practice, especially low grade endometrial stromal sarcoma (ESS) and CL, suggesting the need for novel immunomarkers panels for differential diagnosis. Interferon-induced transmembrane protein 1 (IFITM1) is a novel immunomarker for endometrial stromal cells, h-caldesmon is an immunomarker for smooth muscle cells and has a higher specificity than smooth muscle actin (SMA). So this study aimed to evaluate whether IFITM1, cluster of differentiation 10(CD10), SMA, and h-caldesmon are useful biomarker combinations for the differential diagnosis of EST and CL.

**Methods:**

Tissue microarrays were used to detect IFITM1, CD10, SMA, and h-caldesmon immunohistochemical staining in 30 EST and 33 CL cases.

**Results:**

The expressions of IFITM1 and CD10 were high in EST (86.7 and 63.3%, respectively) but low in CL (18.2 and 21.2%), whereas those of h-caldesmon and SMA were high in CL (87.9 and 100%) and low in EST (6.9 and 40%). In diagnosing EST, IFITM1 shows better sensitivity and specificity (86.7 and 81.8%, respectively) than CD10 (63.3 and 78.8%). The specificity of h-caldesmon in diagnosing CL was significantly higher (93.1%) than that of SMA (60%). When all four antibodies were combined for the differential diagnosis, the area-under-the-curve (AUC) predictive value was 0.995. The best combination for diagnosing EST was IFITM1 (+) or CD10 (+) and h-caldesmon (−) (sensitivity 86.7%, specificity 93.9%).

**Conclusion:**

The best combination for diagnosing CL were h-caldesmon (+) and SMA (+) (sensitivity 87.9%, specificity 100%). IFITM1, CD10, SMA, and h-caldesmon are a good combination for the differential diagnosis of EST and CL.

## Highlights

1. IFITM1 is a novel immunomarker for endometrial stromal cells and tumors
2. It is the first time to combine the four markers for differential diagnosis
3. Our research showed a very helpful and promising result

## Background

Endometrial stromal tumor (EST) is a rare malignant mesenchymal tumor of the uterus. In 2014, the World Health Organization classified EST as endometrial stromal nodule (ESN), low grade endometrial stromal sarcoma (ESS), high grade ESS, and undifferentiated endometrial sarcoma [1]. However, there is an overlap in morphology and immunohistochemistry between EST and leiomyoma, especially for the low grade ESS from cellular leiomyoma (CL). If EST and CL are misdiagnosed, it may lead to overtreatment or undertreatment of the patient, which will affect the survival and prognosis of the patient. Currently, cluster of differentiation 10 (CD10) has been considered as the best immunomarker for endometrial stromal cells [2–6], but it not expressed in all mesenchymal tumors [7–9]. Rather, CD10 is sometimes expressed in leiomyoma [10, 11]. Smooth muscle actin (SMA) is a common biomarker for smooth muscle, however, SMA is sometimes expressed in EST [12–15], suggesting the need for novel immunomarkers and immunohistochemical panels for differentiating between EST and CL.

Interferon-induced transmembrane protein 1 (IFITM1), also called CD225, is a novel immunomarker for endometrial stromal cells and tumors [16, 17] and outperforms CD10 in distinguishing low grade ESS from CL [18, 19]. Meanwhile, h-caldesmon is another immunomarker for smooth muscle cells and has a higher specificity than SMA. Therefore, we suspect that the combined application of IFITM1, CD10, which are mainly expressed in endometrial stromal cells, and the antibodies SMA, and h-caldesmon, which are mainly expressed in smooth muscle, may better help the differential diagnosis of EST and CL. However, there has been no study on the combined use of IFITM1, CD10, SMA, and h-caldesmon in distinguishing between EST and CL. The purpose of this work is to investigate whether IFITM1, CD10, SMA, and h-caldesmon are useful biomarker combinations for the differential diagnosis of EST and CL.

## Methods

### Clinical data

This study enrolled 30 patients with EST (5 with ESNs, 16 with low-grade ESSs, 5 with high-grade ESSs, and 4 with undifferentiated endometrial sarcoma) and 33 patients with CL. Data were collected from 2012 to 2017 from the Department of Pathology of the First Affiliated Hospital of Shihezi University School of Medicine and the Department of Pathology of Xinjiang Uygur Autonomous Region People’s Hospital. All pertinent clinical information was obtained from the hospital electronic medical records. All patients had complete medical history and clinicopathologic data, and all cases were confirmed by surgery and pathology.

### Tissue microarray building

For tumor microarray construction, paraffin-embedded tissues of 63 cases were included as mentioned above [20]. Paraffin blocks and corresponding hematoxylin and eosin (HE)-stained sections were collected, and the HE-stained sections were evaluated by two senior pathologists. Morphologically representative regions were carefully selected on each individual paraffin-embedded block, and a hollow needle (1.0 mm diameter) was used to puncture the selected area to a new small wax block. Considering the specificity of the tumor and the tendency of the paraffin tissue to flake off, two punctures were performed in different areas of each tumor wax block. One section was stained with H&E to evaluate the presence of the tumour by light microscopy.

### Immunohistochemistry

For immunohistochemical analysis, biopsy specimens were fixed in 10% neutral-buffered formalin and routinely processed. The paraffin-embedded blocks were sectioned (4 μm thickness), stained with HE, and observed by microscopy. The two-step immunohistochemical EnVision method was applied. The primary antibodies used were ITIFM1 (Sigma, 1:400), CD10 (ZSGB-BIO, 1:50), SMA (ZSGB-BIO, 1:100) and h-caldesmon (ZSGB-BIO, 1:100). CD10 uses EDTA for antigen retrieval, and all other antibodies use citrate. The staining of IFITM1 and CD10 is located in the cytoplasm and membrane, h-caldesmon and SMA are positive in the cytoplasm. The evaluation of the four biomarkers was assessed twice by two gynecological pathologists with intermediate professional title or above, separated by one-month period. The extent of staining was evaluated as 0%, 0–25%, 26–50%, 51–75%, and 76–100%, and the intensity of staining as absent (0), weak (1+), moderate (2+), and strong (3+). When a different staining evaluation was used, the higher intensity score was used as the final score. The staining score was obtained by multiplying percentage with intensity and this score was used for our statistics analysis. The results were interpreted as described above.

### Statistical analysis

The major purpose of statistical comparison was to seek helpful antibodies to differential diagnosis between EST and CL. First, composition scores for the 4 antibodies tested were determined based on immunohistochemical grades (range 0–12) as intensity (range 0–3) multiplied by percent expression (range 0–4). Then, the expression patterns of the four antibodies were checked, the chi-square test was used to compare the differences between the two groups, and Fisher’s exact test was performed on each marker. The sensitivity, specificity, positive predictive values (PPVs) and negative predictive values (NPVs) were calculated from the screening and diagnostic EST. Among the statistically significant biomarkers, we perform receiver operating characteristic (ROC) curve analysis in descending order, add each biomarker one by one, and use the area under the curve (AUC) to indicate statistical significance [21]. All statistical analyses were performed using SPSS version 17.0. A *p*-value of < 0.05 (all, two-tailed test) was considered as statistically significant.

## Results

### Clinical features

The median age of 30 EST patients was 49.5 (27–73) years, and the main clinical symptoms were irregular vaginal bleeding, abdominal pain, postmenopausal vaginal bleeding, and uterine fibroids. The 33 CL patients had an median age of 41 (26–60) years and mainly showed clinical manifestations of dysmenorrhea, prolonged menstrual period, and increased menstrual volume.

**Fig. 1:**
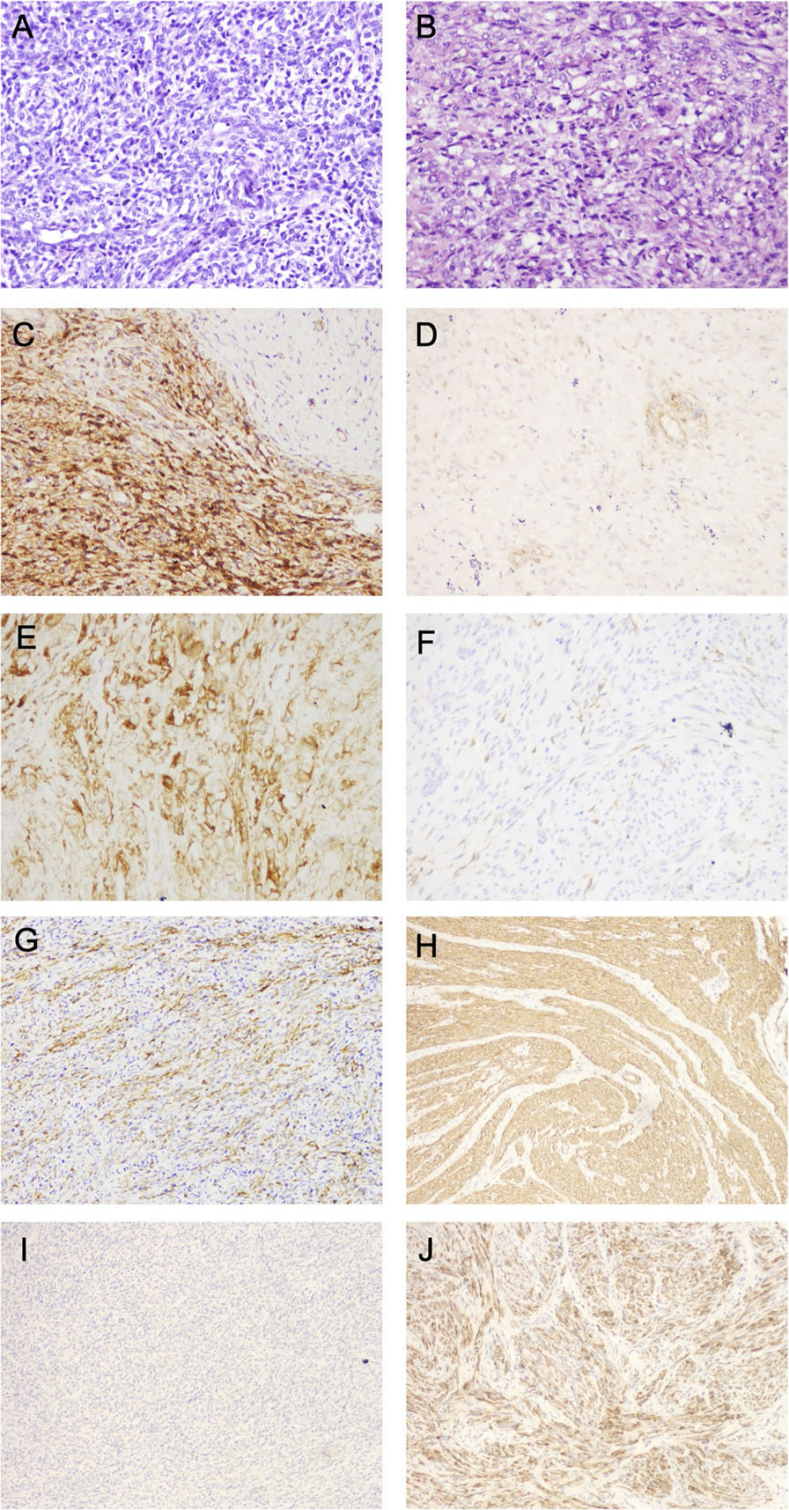
Immunohistochemical results. **a** Endometrial stromal Tumors (hematoxylin and eosin stains, magnification 200). **b** Cellular leiomyomas (hematoxylin and eosin stains, magnification ×200). **c** Endometrial stromal tumors showing strong positive results for IFITM1. **d** Cellular leiomyomas showing a negative or weak expression of IFITM1. **e** Endometrial stromal tumors exhibiting a positive expression of CD10. **f** Cellular leiomyomas exhibiting a weekly CD10 positivity. **g** Endometrial stromal tumors demonstrating SMA reactivity. **h** Cellular leiomyomas showing strong positive results for SMA. **i** Endometrial stromal tumors showing a negative or weak expression of h-caldesmon. **j** Cellular leiomyomas demonstrating strong positive results for h-caldesmon

**Fig. 2:**
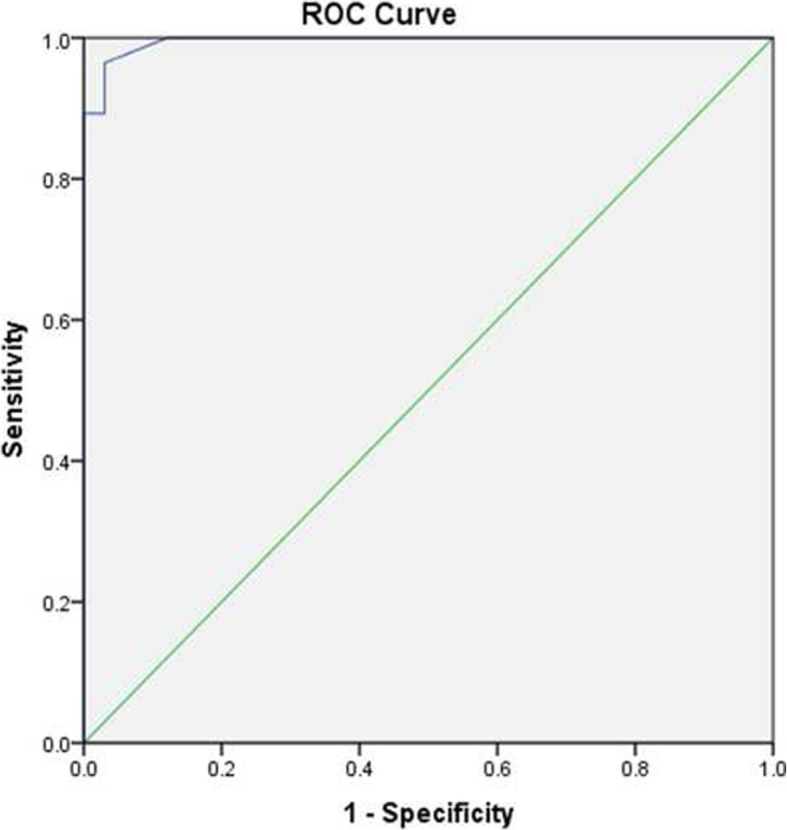
Receiver operating characteristic curve for prediction of endometrial stromal tumors and cellular leiomyoma. Comparison of EST (*n* = 30) and uterine cellular leiomyomas (CL) (*n* = 33). All 4 markers include IFITM1, CD10, SMA and h-caldesmon

The immunohistochemical results are summarized in Table 1 and illustrated in Fig. 1. The ROC values, sensitivity, specificity, PPVs, and NPVs are summarized in Tables 2, 3, 4 and 5 and shown in Fig. 2.

**Table 1:** Intensity of immunohistochemical staining of IFITM1, CD10, h-caldesmon and SMA in endometrial stromal tumor and cellular leiomyoma

Antibodies	EST (30 cases)					CL (33 cases)				
	Positive	Category Intensity				Positive	Category Intensity			
		0	1+	2+	3+		0	1+	2+	3+
IFITM1	26 (86.7%)	4	10	8	8	6 (18.2%)	27	6	0	0
CD10	19 (63.3%)	11	6	7	6	7 (21.2%)	26	7	0	0
h-caldesmon	2 (6.7%)	28	2	0	0	29 (87.9%)	4	15	10	4
SMA	12 (40%)	18	4	6	2	33 (100%)	0	22	9	2

*Abbreviations*: *EST* indicates Endometrial Stromal Tumor, *CL* indicates Cellular Leiomyoma

**Table 2:** Sensitivity, Specificity, Positive Predictive Value and Negative Predictive Value of IFITM1 and CD10 for endometrial stromal tumor and h-caldesmon and SMA for cellular leiomyoma

Tumor	Antibodies	Sensitivity (%)	Specificity(%)	PPV (%)	NPV (%)
EST	IFITM1	86.7	81.8	81.3	87.1
	CD10	63.3	78.8	73.1	70.3
CL	h-caldesmon	87.9	93.3	93.5	87.5
	SMA	100.0	60.0	73.3	100.0

*Abbreviations*: *EST* indicates Endometrial Stromal Tumor, *CL* indicates Cellular Leiomyoma, *PPV* indicates Positive Predictive Value, *NPV* indicates Negative Predictive Value

**Table 3:** Using receiver operating characteristic curves to evaluate the area-under-the-curve predictive value for prediction of endometrial stromal tumor and cellular leiomyoma

Groups for prediction of EST and CL	AUC
Combination of IFITM1 and CD10	0.930
Combination of IFITM1 and h-caldesmon	0.976
Combination of IFITM1and SMA	0.907
Combination of CD10 and h-caldesmon	0.952
Combination of CD10 and SMA	0.830
Combination of IFITM1, CD10, and h-caldesmon	0.984
Combination of IFITM1, CD10, and SMA	0.980
Combination of CD10, SMA, and h-caldesmon	0.956
Combination of IFITM1, CD10, SMA, and h-caldesmon	0.995

*Abbreviations*: *AUC* indicates Area-under-the-curve predictive value, *EST* indicates Endometrial Stromal Tumor, *CL* indicates Cellular leiomyoma

**Table 4:** The sensitivity and specificity of combined IFITM1, CD10, h-caldesmon and SMA immunostaining in the diagnosis of endometrial stromal tumor

Groups	Sensitivity (%)	Specificity (%)
IFITM1 (+) and h-caldesmon (−) for EST	80.0	100.0
IFITM1 (+) and SMA (−) for EST	60.0	97.0
CD10 (+) and h-caldesmon (−) for EST	50.0	100.0
CD10 (+) and SMA (−) for EST	36.7	100.0
IFITM1 (+) and CD10 (+) for EST	56.7	93.9
IFITM1 (+) or CD10 (+) for EST	93.3	66.7
IFITM1 (+) or CD10 (+) and h-caldesmon (−) for EST	86.7	93.9
IFITM1 (+) and CD10 (+) and h-caldesmon (−) for EST	53.3	100.0
IFITM1 (+) or CD10 (+) and SMA (−) for EST	56.7	100.0
IFITM1 (+) and CD10 (+) and SMA (−) for EST	30.0	100.0
IFITM1 (+) and CD10 (+) and h-caldesmon (−) and SMA (−) for EST	31.0	100.0
IFITM1 (+) or CD10 (+) and h-caldesmon (−) and SMA (−) for EST	56.7	100.0

*Abbreviations*: *EST* indicates Endometrial Stromal Tumor

**Table 5:** The sensitivity and specificity of combined IFITM1,CD10, h-caldesmon and SMA immunostaining in the diagnosis of cellular leiomyoma

Groups	Sensitivity (%)	Specificity (%)
h-caldesmon (+) and IFITM1 (−) for CL	69.7	100.0
SMA (+) and IFITM1 (−) for CL	81.8	93.1
h-caldesmon (+) and CD10 (−) for CL	72.7	96.6
SMA (+) and CD10 (−) for CL	78.8	82.8
h-caldesmon (+) and SMA (+) for CL	87.9	100.0
h-caldesmon (+) or SMA (+) for CL	100.0	57.1
h-caldesmon (+) and SMA (+) and IFITM1 (−) for CL	69.7	100.0
h-caldesmon (+) or SMA (+) and IFITM1 (−) for CL	81.8	93.1
h-caldesmon (+) and SMA (+) and CD10 (−) for CL	72.7	96.6
h-caldesmon (+) or SMA (+) and CD1 (−) for CL	78.8	82.8
h-caldesmon (+) and SMA (+) and IFITM1 (−) and CD10 (−) for CL	57.6	100.0
h-caldesmon (+) or SMA (+) and IFITM1 (−) and CD10 (−) for CL	66.7	93.1

*Abbreviation*: *CL* indicates Cellular leiomyoma

### IFITM1 and CD10

Both EST (Fig. 1a) and CL cases (Fig. 1b) showed a dense spindle-cell braid-like arrangement. Among the 30 EST cases, 26 (86.7%) demonstrated IFITM1 cytoplasmic positivity (Fig. 1c). The staining intensity was strong (3+) in 8 cases, moderate (2+) in 8 cases, and weak (1+) in 10 cases. Of the 33 CL cases, only 6 (18.2%) demonstrated IFITM1 nuclear positivity (Fig. 1d), all of which scored weak (1+) in intensity. CD10 was expressed in 19 (63.3%) of the 30 EST cases (Fig. 1e). The staining in these cases occurred in the cell cytoplasm and was strong (3+) in 6 cases, moderate (2+) in 7 cases, and weak (1+) in 6 cases. Only 7 (21.2%) of the 33 CL cases were CD10(+), and all positive cases had a weak (1+) intensity (Fig. 1f).

### SMA and h-caldesmon

SMA was positive in 12 (40%) of the 30 EST cases (Fig. 1g). The staining in these cases was expressed in the cytoplasm and was moderate to strong (2+ to 3+) in 8 cases and weak (1+) in 4 cases. All 33 (100%) CL cases expressed SMA (Fig. 1h), and among them, the staining was moderate to strong (2+ to 3+) in 11 cases and weak (1+) in the remaining cases. Meanwhile, h-caldesmon was expressed in the cell cytoplasm of only 2 (6.7%) of the 30 EST cases (Fig. 1i), and the staining in these positive cases were weak (1+). However, 29 (87.9%) of the 33 CL cases exhibited h-caldesmon positivity (Fig. 1j). In these 33 CL cases, the staining was strong (3+) in 4 cases, moderate (2+) in 10 cases, and weak (1+) in 15 cases.

### Comparison of the expression of IFITM1, CD10, h-caldesmon, and SMA in endometrial stromal tumor and cellular leiomyoma between pre- and post-menopausal women

In order to avoid the influence of hormones on tumor expression, combined with clinical information, we divided the patients into two groups of pre- and post-menopausal women, and compared the expression of IFITM1, CD10, h-caldesmon, and SMA in EST and CL between pre- and post-menopausal women (Table 6). The results showed that in the same tumor, the expressions of IFITM1, CD10, h-caldesmon, and SMA were not statistically different between pre- and post-menopausal groups. In the two groups of pre- and post-menopausal, the expressions of IFITM1, SMA, and h-caldesmon were significantly different in EST and leiomyomas and showed the same trend. CD10 was slightly different, and its expression was significant difference in premenopausal EST and CL, but there was no statistical difference in post-menopausal group. From the above results, we believe that hormones have no significant effect on the expression of tumor antibodies.

**Table 6:** Comparison of the expression of IFITM1, CD10, h-caldesmon, and SMA in endometrial stromal tumor and cellular leiomyoma between pre- and post-menopausal women

Antibody	Tumor	Menopausal	Positive	Negative	*P* value
IFITM1	EST	pre	17	2	
		post	9	2	0.552
CD10		pre	13	6	
		post	4	7	0.088
SMA		pre	12	7	
		post	6	5	0.643
h-caldesmon		pre	2	17	
		post	0	11	0.265
IFITM1	CL	pre	5	23	
		post	1	4	0.909
CD10		pre	6	22	
		post	0	5	0.252
SMA		pre	28	0	
		post	5	0	/
h-caldesmon		pre	24	4	
		post	5	0	0.367
Antibody	Women	Tumor	Positive	Negative	*P* value
IFITM1	Pre-menopausal	ESTs	17	2	
		CL	5	23	0
CD10		ESTs	15	7	
		CL	4	21	0
SMA		ESTs	6	13	
		CL	28	0	0
h-caldesmon		ESTs	2	17	
		CL	24	4	0
IFITM1	Post- menopausal	ESTs	9	2	
		CL	1	4	0.018
CD10		ESTs	4	7	
		CL	0	5	0.119
SMA		ESTs	5	6	
		CL	5	0	0.037
h-caldesmon		ESTs	0	11	
		CL	5	0	0

*Abbreviations*: *EST* indicates Endometrial Stromal Tumor, *CL* indicates Cellular leiomyoma; “/” indicates no *P* value

### Sensitivity, specificity, positive predictive values, and negative predictive values of IFITM1, CD10, h-caldesmon, and SMA

In the diagnosis of EST, IFITM1 showed a sensitivity of 86.7%, a specificity of 81.8%, a PPV of 81.3%, and an NPV of 87.1%. For CD10, the sensitivity, specificity, PPV, and NPV were 63.3, 78.8, 73.1, and 70.3%, respectively. h-caldesmon positivity may support a diagnosis of CL, showing a sensitivity of 87.9%, a specificity of 93.3%, a PPV of 93.5%, and an NPV of 87.5%. SMA had the highest sensitivity (100%), but its specificity was 60%, significantly lower than that of h-caldesmon. SMA had a PPV and an NPV of 73.3 and 100%, respectively (Table 2).

### IFITM1, CD10, h-caldesmon, and SMA as a useful combination for differential diagnosis

Based on the expressions of the four antibodies and their ROC curve, the combination of IFITM1, CD10, SMA, and h-caldesmon four antibodies showed the highest predictive value of AUC, and the ROC values of other combinations are lower than this type of combination (Table 3, Fig. 2), we speculate that their combinations could be helpful in the differential diagnosis of EST and CL.

When all four antibodies were combined for the EST diagnosis (Table 4), The three most sensitive combinations in descending order were IFITM1 (+) or CD10 (+), IFITM1 (+) or CD10 (+) and h-caldesmon (−), IFITM1 (+) and h-caldesmon (−), with their sensitivity of 93.3, 86.7, 80%, respectively. The combination of antibodies greatly increased the specificity of EST diagnosis, the specificity of combinations of IFITM1 (+) and h-caldesmon (−), IFITM1 (+) and CD10 (+) and h-caldesmon (−) and SMA (−), and IFITM1 (+) or CD10 (+) and h-caldesmon (−) and SMA (−) were 100%. Considering both sensitivity and specificity, the combination with the best diagnostic value for EST was IFITM1 (+) or CD10 (+) and h-caldesmon(−), with a sensitivity and a specificity of 86.7 and 93.9%, respectively.

In diagnosing CL (Table 5), the three most sensitive combinations in descending order were h-caldesmon (+) or SMA (+), h-caldesmon (+) and SMA (+), and h-caldesmon (+) or SMA (+) and IFITM1 (−), with sensitivity values of 100, 87.9, and 81.8%, respectively. On the other hand, h-caldesmon (+) and IFITM1 (−), h-caldesmon (+) and SMA (+), h-caldesmon (+) and SMA(+) and IFITM1 (−) showed better specificity for predicting CL from EST with all specificity were 100%. Taking into account sensitivity and specificity, h-caldesmon (+) and SMA (+) was the best combination for distinguishing CL from EST, with a sensitivity of 87.9% and a specificity of 100%. The second-best combination for distinguishing CL from EST was h-caldesmon (+) or SMA (+) and IFITM1 (−), with a sensitivity of 81.8% and a specificity of 93.1%.

## Discussion

Distinguishing EST from CL, especially low grade ESS from CL is always a problem. Finding an effective combination of immunohistochemistry can provide help for the differential diagnosis of EST and CL. The standard convention immunomarker panel used by most pathologists to distinguish EST from CL consists of CD10, h-caldesmon, and SMA [10, 22–24], and an immunoprofile of CD10 (+), h-caldesmon (−), and SMA (−) supports the diagnosis of EST [15]. However, the current combination of immunohistochemical antibodies has been shown to be inaccurate, especially when diagnosing EST using CD10 alone [3, 10]. CD10 is not merely expressed in EST but is also positively expressed in 20–30% of smooth muscle tumors [13, 15]. SMA is a common muscle marker for EST and therefore has a very low specificity. Although h-caldesmon has a higher specificity that of SMA, its sensitivity is worse [10, 13, 15, 25]. Thus, the need for a novel biomarker or a new immunohistochemical combination is imperative.

IFITM1 is a novel biomarker for endometrium stromal cells and is reported to be more valuable than CD10 [19, 26]. According to Busca et al. [19], IFITM1 and CD10 were expressed in 14 ESS cases, and although their sensitivities were 83 and 91%, respectively, IFITM1 showed a higher specificity than CD10, that is, 70% vs 45%. These findings are consistent with our findings, which state that IFITM1 was more specific and sensitive than CD10 in EST (sensitivity 86.7% vs. 63.3%, specificity 81.8% vs. 78.8%). However, the author only compared the expression of CD10 and IFITM1, moreover, they merely collected 14 cases. Rush et al. [13] compared the expressions of SMA and h-caldesmon between EST and CL and found that SMA was more sensitive than h-caldesmon (90.9% vs. 72.7%); but, h-caldesmon was more specific than SMA (100% vs. 91.7%). However, the author did not study CD10 and focused on myogenic markers only. In our study, h-caldesmon showed a lower sensitivity than SMA (87.9% vs. 100%), but its specificity was significantly higher (93.3% vs. 60%).

In general, no one immunomarker is sensitive and specific enough to make an accurate diagnosis. Therefore, surgical pathologists usually run an immunohistochemical antibody panel to help them diagnose challenging cases. Based on the expressions of the four antibodies and their ROC curve (the AUC predictive value was 0.995), we speculate that their combination could be useful in the clinical and differential diagnosis of EST and CL. We found that the best panel for diagnosing EST was IFITM1 (+) or CD10 (+) and h-caldesmon (−) (sensitivity 86.7%, specificity 93.9%). Though the combination of IFITM1(+) or CD10(+) had higher sensitivity (93.3% VS 86.7%) and the specificity of many other combinations reached 100%. The best combination for diagnosing CL were h-caldesmon (+) and SMA (+) (sensitivity 87.9%, specificity 100%), nevertheless the combination of h-caldesmon(+) or SMA(+) had the highest sensitivity (100% VS 87.9%), with its specificity only 57.1%.

However, there are certain limitations in that the ROC curve cannot completely show the positive and negative expressions of the antibodies. Because high grade ESS is rare and the number of samples is not enough, we could not compare low grade ESS with high grade ESS, so we focused on the differential diagnosis between low grade ESS and CL. No literature had reported the combination of these four biomarkers. In short, our research provides a useful combination of immunological markers for the differential diagnosis of ESTs and CLs with similar morphology, and helps pathologists make accurate diagnoses to guide treatment.

## Conclusion

This study revealed that the combination of IFITM1, CD10, SMA, and h-caldesmon comprised the best immunohistochemical panel for differentiating between EST and CL, especially when the clinical history and histological morphology cannot be differentiated totally. Considering the costs, we also recommend the combinations IFITM1 and h-caldesmon for the same purpose. Furthermore, future validation in distinguishing ESS from CL, particularly low grade ESS and CL, as this is a more difficult differentiation for pathologists.

## Data Availability

The datasets used and/or analyzed during the current study are available from the corresponding author on reasonable request.

## Abbreviations

EST: Endometrial stromal tumor
CL: Cellular leiomyoma
ESN: Endometrial stromal nodule
ESS: Endometrial stromal sarcoma
IFITM: Interferon-induced transmembrane protein 1
CD10: Cluster of differentiation 10
SMA: Smooth muscle actin
HE: Hematoxylin-eosin staining
PPV: Positive predictive value
NPV: Negative predictive value
ROC curve: Receiver operating characteristic curve
AUC: Area-under-the-curve
“/”: No *P* value
